# A simple quantum voting scheme with multi-qubit entanglement

**DOI:** 10.1038/s41598-017-07976-1

**Published:** 2017-08-08

**Authors:** Peng Xue, Xin Zhang

**Affiliations:** 1 Department of Physics, Southeast University, Nanjing, 211189 China; 2 State Key Laboratory of Precision Spectroscopy, East China Normal University, Shanghai, 200062 China

## Abstract

We propose a simple quantum voting scenario with a set of pairs of particles in a multi-particle entangled state. This scenario is suitable for large scale general votings. We also provide a proof of security of our scheme against the most general type of attack by generalizing Shor and Preskill’s proof of security of the other schemes.

## Introduction

Cryptography is the art of rendering a message unintelligible to any unauthorized party. Over one century, many applications of classical cryptography have been discovered. One of the most outstanding applications is secure voting. Classical secret voting protocols have been investigated by many researchers from both the practical and theoretical points of view^1–9^. Let us briefly review the goal of a secure voting scheme. Usually, a classical secure voting scheme requires the following conditions: 1 Only the votes of the authorized voters can be taken into account; 2 Every interested voter must vote exactly once. Voting more than once can not be accepted by the administrator and other voters; 3 No one can control other voters’ opinions about the issues to be voted upon; 4 No one can duplicate the votes of the other voters from being detected by the administrator and other voters. For any classical voting scheme, the electronic votes can be easily duplicated; 5 No one can change the votes of the other voters from being detected by the administrator and the other voters; 6 Every voter can confirm that her/his vote is taken into account by the administrator. In addition, sometimes, secret ballot is needed.

Unfortunately, security of classical cryptography is based on the unproven complexity of calculating certain functions, such as the difficulty of factoring large integers. However, the recent work in quantum computation^10^ shows that quantum computers can, at least in principle, factor much faster than classical computers, which means that classical cryptography is already insecure.

The security of quantum cryptography is based on the fundamental postulate of quantum physics that “every measurement perturbs a system”. Indeed, passive monitoring of transmitted signals is strictly forbidden in quantum mechanics. The “quantum no-cloning theorem^11, 12^’’ indicates that it is impossible to make an exact copy if an unknown quantum state.

It is known that for some tasks such as bit commitment, quantum mechanics can not help^13, 14^. However for others, such as quantum voting, a number of novel quantum protocols have been developed^7, 15–22^. Quantum cryptography brings an entirely new way of solving the secure voting problem.

In this paper, we propose a simple quantum voting scheme for world environments with the following conditions: (a) This protocol involves voters and an administer who also plays a role as a counter, and a scrutinizer. If the issue of votes are controlled by a single administrator, sh/he may add extra votes as she/he wished. To overcome this problem, the whole election should be monitored by a scrutinizer in our protocol; (b) The six conditions required in classical secure voting are satisfied in our protocol; (c) The computations among voters are independent without the requirement of any global computation and a voter only has to communicate with the scrutinizer and finally sends his vote to the counter (administrator). So this protocol is suitable for large scale general elections; (d) Its security is guaranteed by the fundamental postulate of quantum physics; (e) The physical requirements of the scheme well fit the current experimental technique. The validity of the result is base on the assumption that one of the administrator and scrutinizer is absolutely trusted at least.

## Results

Suppose that Alice is one of the voters, and there are an administrator named Bob and a scrutinizer named Carol which are equal in status and supervise each other’s performance. If one of them wants to cheat in the election, it can be detected by the other. Carol prepares *M* pairs of particles in Greenbergee-Horne-Zeilinger (GHZ) type of maximally entangled state

1 $$\begin{array}{rcl}|\phi \rangle  & = & \frac{1}{\sqrt{2}}\mathrm{(|000000}\rangle +\mathrm{|111111}\rangle )\\  & = & \frac{1}{(2\sqrt{2})}\mathrm{(|000}\rangle +\mathrm{|111}\rangle {)}_{123}\mathrm{(|000}\rangle +\mathrm{|111}\rangle {)}_{456}\\  &  & +\frac{1}{(2\sqrt{2})}\mathrm{(|000}\rangle -\mathrm{|111}\rangle {)}_{123}\mathrm{(|000}\rangle -\mathrm{|111}\rangle {)}_{456}\mathrm{.}\end{array}$$

Particles 1 is sent to Bob, particles 2 and 3 are sent to Alice and particles 4, 5 and 6 are left for Carol herself. Alice receives the particles from Carol and encodes her opinion on the vote, which is represented in binary notation as *a* ∈ {000, 001, …, 111}, by performing the operation *B*_*b*_ ⊗ *C*_*c*_ corresponding to *b* = *g*(*a*) on them (See Table 1), where *g*(*x*) is bijection.

**Table 1:** Here, initially the particles 1, 2 and 3 are in the GHZ state ${|\psi \rangle }_{i}=\frac{1}{\sqrt{2}}$(|000〉 ± |111〉 which is determined by the result of the GHZ measurement on the rest qubits 4, 5 and 6. After a certain operation, the three qubits will be in the final state ${|\psi \rangle }_{f}$ if *σ*_*x*_, *σ*_*y*_ and *σ*_*z*_ are Pauli operators.

*b*_*l*_	${{\boldsymbol{B}}}_{{\boldsymbol{bl}}}^{{\bf{2}}}$	${{\boldsymbol{C}}}_{{\boldsymbol{bl}}}^{{\bf{3}}}$	${|{\boldsymbol{\psi }}\rangle }_{{\boldsymbol{f}}}={{\boldsymbol{I}}}^{{\bf{1}}}\otimes {{\boldsymbol{B}}}_{{{\boldsymbol{b}}}_{{\boldsymbol{l}}}}^{{\bf{2}}}\otimes {{\boldsymbol{C}}}_{{{\boldsymbol{b}}}_{{\boldsymbol{l}}}}^{{\bf{3}}}{|{\boldsymbol{\psi }}\rangle }_{{\boldsymbol{i}}}$
0	I	I	$\frac{1}{\sqrt{2}}(|000\rangle \pm |111\rangle )$
1	I	*σ*_*x*_	$\frac{1}{\sqrt{2}}(|001\rangle \pm |110\rangle )$
2	*σ*_*z*_	I	$\frac{1}{\sqrt{2}}(|000\rangle \mp |111\rangle )$
3	*σ*_*z*_	*σ*_*x*_	$\frac{1}{\sqrt{2}}(|000\rangle \mp |111\rangle )$
4	*σ*_*x*_	I	$\frac{1}{\sqrt{2}}(|010\rangle \pm |001\rangle )$
5	*σ*_*x*_	*σ*_*x*_	$\frac{1}{\sqrt{2}}(|011\rangle \pm |100\rangle )$
6	−*iσ*_*y*_	I	$\frac{1}{\sqrt{2}}(|010\rangle \mp |101\rangle )$
7	−*iσ*_*y*_	*σ*_*x*_	$\frac{1}{\sqrt{2}}(|011\rangle \mp |100\rangle )$

Remarkably, in order to keep the votes from being duplicated, the voters perform the voting process with probability 1 − *ε* and the detecting process with probability *ε*, where 0 < *ε* < 1.

Suppose there are *n* candidates to be voted, which are coded in binary notation. In the voting process, Alice sends the two resulting particles (three bits of information) representing her opinion on vote to Bob every time. This kind of communication has be done for *N* times in all. Then the vote from Alice can be represented as $\upsilon =({\sum }_{l=1}^{N}{a}_{l})\,{\rm{mod}}\,n$.

The voters, administrator and scrutinizer are connected by a quantum channel and a classical public channel. They follow the below procedures.

1 With probability 1 − *ε*, Alice performs the voting process. She randomly chooses a coding scheme *g*: {0, 1, …, 7}→ {*B*_0_ ⊗ *C*_0_, *B*_1_ ⊗ *C*_1_, …, *B*_7_ ⊗ *C*_7_}.

Suppose this is the *l* th part of opinion on her vote. She encodes the value by performing the operation corresponding to *b*_*l*_ = *g*(*a*_*l*_) on the particles 2 and 3. Even if there is any eavesdropper Eve who tries a “man-in the middle” attack^23^ to duplicate the vote finds out which operation Alice has performed, she still does not know her message. The coding scheme *g* acts as a secret key used by Alice. Here, there are eight unitary operations ${B}_{{b}_{l}}\otimes {C}_{{b}_{l}}$ (seeing Table 1) on the particles 2 and 3. This process is similar to the scheme of densecoding using multiparticle quantum channel^24^.

Then, she sends the resulting particles 2 and 3 to the administrator Bob.

2 At the same time, Carol makes a GHZ measurement on the particles 4, 5 and 6 and announces the result over the public channel. Then, she sends the particle 6 to Alice, and the rest particles are left for herself.

3 Bob receives Particles 2 and 3 and measures the three qubits resulting in the final state |*ψ*〉_*f*_ if along the GHZ basis. According to the result of Carol’s measurement, he learns the form of the operation ${B}_{{b}_{l}}\otimes {C}_{{b}_{l}}$ (that is *b*_*l*_), but not the three bits of information representing one part of Alice’s opinions about the issues to be voted upon.

4 In the meantime of Step 1, with probability *ε*, Alice performs the detecting process. She measures the particle 2 along the orthogonal basis $\{|+\rangle =\frac{1}{\sqrt{2}}(|0\rangle +\mathrm{|1}\rangle ),|-\rangle =\frac{1}{\sqrt{2}}\mathrm{(|0}\rangle -\mathrm{|1}\rangle )\}$. The particles 1 and 3 will be in one of the two EPR states |Φ±〉 = $\frac{1}{\sqrt{2}}$(|00〉±|11〉) with the same probability determined by both of the results of the measurement of Carol and Alice. Alice randomly chooses one out of the rectilinear ({|0〉, |1〉}) and orthogonal bases and measures particle 3 along it. Then she announces the two results of her measurement and the basis she has chosen.

5 If Bob has received the information over the public channel (not the particles), he will also perform the detecting process. He measures his particle 1 along the same basis and detects the correlations of the particles 1, 2 and 3. For example, if Carol measure the particles 4, 5 and 6 and gets the state |*ψ*〉_456_ = $\frac{1}{\sqrt{2}}$(|000〉 + |111〉)_456_, the rest particles 1, 2 and 3 will be in the same state |*ψ*〉_123_ = $\frac{1}{\sqrt{2}}$(|000〉 + |111〉)_123_. If the result of Alice’s measurement is $\frac{1}{\sqrt{2}}$(|0〉 − |1〉), the results of correlation detection are shown in Table 2. If the number of the mismatches *r* (here, mismatch means the results of Alice and Bob are not correlated), the error rate of the channel is estimated as *e* = (*r*)/(*m*) (*m* is the number of the pairs of particles used to detect the security of the quantum channel).

2 $$m=\varepsilon M\mathrm{.}$$

**Table 2:** Example of the detecting process.

|*ψ*〉_456_ (|*ψ*〉_123_)	$$\frac{1}{\sqrt{2}}(| 000\rangle +| 111\rangle )$$	$$\frac{1}{\sqrt{2}}(| 000\rangle +| 111\rangle )$$	$$\frac{1}{\sqrt{2}}(| 000\rangle +| 111\rangle )$$	$$\frac{1}{\sqrt{2}}(| 000\rangle +| 111\rangle )$$
result 1 (Alice)	$\frac{1}{\sqrt{2}}(|0\rangle -|1\rangle )$	$\frac{1}{\sqrt{2}}(|0\rangle -|1\rangle )$	$\frac{1}{\sqrt{2}}(|0\rangle -|1\rangle )$	$\frac{1}{\sqrt{2}}(|0\rangle -|1\rangle )$
|*ϕ*〉_13_	|Φ^−^〉	|Φ^−^〉	|Φ^−^〉	|Φ^−^〉
basis(Alice)	{|0〉, |1〉}	{|0〉, |1〉}	{|+〉, |−〉}	{|+〉, |−〉}
result 2 (Alice)	|0〉	|1〉	$|+\rangle$	$|-\rangle$
result 3 (Bob)	|0〉	|1〉	$|-\rangle$	$|+\rangle$

Here {|+〉 = $\frac{1}{\sqrt{2}}$(|0〉 + |1〉), |−〉 = $\frac{1}{\sqrt{2}}$(|0〉−|1〉)} represents the  +  |Φ^−^〉 = $\frac{1}{\sqrt{2}}$(|00〉 − |11〉).

Note that the test samples *m* are sufficiently large, the estimated error rates *e* should be rather accurate. Now they demand that *e* < *e*_*max*_ where *e*_*max*_ is a prescribed maximally tolerable error rate. In our scheme, after Alice measures her particles with the orthogonal basis, the detection process is equal to that of a modified EPR scheme^25, 26^. Please see refs 26–28 for details. If the constraint is satisfied, it has been proved that the error rate of the signal is also small enough to allow the stabilizer code to correct. Then they proceed to the next steps. Otherwise, they re-start the whole procedure.

6 Then Alice announces the coding scheme *g*, that is the relation among *a*_*l*_, *b*_*l*_ and the corresponding operations.

7 Bob calls out the vote, but the other voters apart from the scrutinizer do not know who has sent this vote.

8 Since Carol and Alice share another GHZ state |*ψ*〉_456_, she can send a return receipt to Alice and make her to confirm her vote is taken into the results of the election. This step is achieved as Alice just has done. Carol performs the process to send receipt with probability 1 − *ε* as the modified densecoding using GHZ channel (seeing Steps 1 and 3). Or, she may perform the detecting process with *ε* to ensure the security of the channel (seeing Steps 5 and 6).

Since the security of our protocol is proven in Step 5, here, we show a data analysis guarantees the security of this protocol against a “man-in the middle” strategy^23^. An eavesdropper Eve intercepts and catches the two particles from Carol and prepares another GHZ state |*ψ* ^*e*^〉_123_ = $\frac{1}{\sqrt{2}}$(|000〉 + |111〉)_123_, and sends two particles to Alice. Then she catches the resulting qubits and makes a GHZ measurement on them to obtain the operation Alice has performed. She put the same operation on the initial particles which she has intercepted from Carol and sends them to Bob. Such an attack is called a “man-in-the-middle” attack.

If Eve is really smart she will try a man-in-the middle attack and duplicate the votes from the authorized voters. However, the voter will perform the detecting process with probability *ε*. So Eve has no chance to adapt her strategy. Then, an error rate is introduced showing the wrong type of correlation in the detecting process with probability $\frac{1}{4}$. That is with probability $\frac{1}{4}$ Eve is detected. Since the probability for a detecting process is *ε*, for *M* pairs of particles, there are *m* pairs of particles are used to detect the security of channel (seeing Eq. 2). If Eve attacks all the time, the probability that she will not be detected with probability *P*_*d*_.

3 $${P}_{d}={(\frac{3}{4})}^{m}\mathrm{.}$$

which is goes to 0 as *m* is large enough.

Except for “man-in-the middle” attack, we will analyze the protocol against some other outsider’s attack, such as “entangle-and-measure attack”. Assume Eve applies the entangled state attack strategy. Namely, Eve takes an attack strategy by applying an arbitrary operation on her own ancillary state and the ballot state (particles 1, 2 and 3) which entangles the ancilla and the particles 1, 2, and 3. Her intervention can then be detected by the administrator Bob, which implies that Eve cannot change the ballot results of voters without being detected. Suppose Eve tries to attack the scheme by entangling her own particle as an ancilla with the ballot state. After voting, the administrator Bob may notice the entangled state which is shared with Alice previously is changed in Step 5. He then sends the states to corresponding voters to detect the destroyed votes. Therefore, whether or not Eve casts to the ballot state, the result can always be detected by voter Alice, which implies that Eve cannot intervene the procession of the ballot. Furthermore, the protocol can be further improved by introducing decoy state. To counter this attack, the decoy states are randomly inserted and when they are examined for security, the total detection probability of Eve is decreased by taking into account that the probability of generation of each decoy state.

There are still many questions that remain open and deserve further detailed investigation. Primarily, it is the analysis of the security of quantum voting against more sophisticated attacks, such as collaborating parties, the use of illegal voting operations, and cheating authorities that deserve further attention.

## Discussion

Quantum secure voting could also be realized by using quantum key distribution. Each authorized voter encrypts her/his vote using quantum key, and sends it to the administrator who shares the key. Then, she/he decrypts the encrypted message and obtains the vote. Since this protocol needs a multi-party quantum key distribution between the administrator and each voter at first, it will bring vast waste of quantum resource. In our protocol, the voting is a deterministic process and does not need either a quantum key distribution or a teleportation compared to the previous protocols. There are one administrator and scrutinizer which supervise each other’s performance and prevent other one from cheating. It is needed that one of them is absolutely trusted at least. We also prove that the protocol is secure against some outsider’s attacks such as man-in-the-middle and entangle-and-measure attacks. Since the computations among voters are independent without the need of any global computation and every voter only has to communicate with the scrutinizer and finally sends his vote to the administrator, this protocol is suitable for large scale general elections and allows to be within the reach of current technology.
